# Preclinical Studies of the Off-Target Reactivity of AFP_158_-Specific TCR Engineered T Cells

**DOI:** 10.3389/fimmu.2020.00607

**Published:** 2020-04-27

**Authors:** Lun Cai, Leidy D. Caraballo Galva, Yibing Peng, Xiaobing Luo, Wei Zhu, Yihong Yao, Yun Ji, Yukai He

**Affiliations:** ^1^Georgia Cancer Center, Medical College of Georgia, Augusta, GA, United States; ^2^The Graduate School, Augusta University, Augusta, GA, United States; ^3^Cellular Biomedicine Group (CBMG), Gaithersburg, MD, United States; ^4^Department of Medicine, Medical College of Georgia, Augusta University, Augusta, GA, United States; ^5^Department of Biochemistry and Molecular Biology, Medical College of Georgia, Augusta University, Augusta, GA, United States

**Keywords:** T cell receptors, T cell engineering, alpha fetoprotein, hepatocellular carcinoma, TCR cross-reactivity, off-target toxicity, immunotherapy

## Abstract

Autologous T cells engineered with T receptor genes (TCR) are being studied to treat cancers. We have recently identified a panel of mouse TCRs specific for the HLA-A0201/alpha fetoprotein epitope (AFP_158_) complex and have shown that human T cells engineered with these TCR genes (TCR-Ts) can eradicate hepatocellular carcinoma (HCC) xenografts in NSG mice. However, due to TCR’s promiscuity, their off-target cross-reactivity must be studied prior to conducting clinical trials. In this study, we conducted *in vitro* X-scan assay and *in silico* analysis to determine the off-target cross-reactivity of 3 AFP_158_-specific TCR-Ts. We found that the 3 AFP_158_-specific TCR-Ts could be cross-activated by ENPP1_436_ peptide and that the TCR3-Ts could also be activated by another off-target peptide, RCL1_215_. However, compared to AFP_158_, it requires 250 times more ENPP1_436_ and 10,000 times more RCL1_215_ peptides to achieve the same level of activation. The EC_50_ of ENPP1_436_ peptide for activating TCR-Ts is approximately 17–33 times higher than AFP_158_. Importantly, the ENPP1+ tumor cells did not activate TCR1-Ts and TCR2-Ts, and only weakly activated TCR3-Ts. The IFNγ produced by TCR3-Ts after ENPP1+ cell stimulation was >22x lower than that after HepG2 cells. And, all TCR-Ts did not kill ENPP1 + tumor cells. Furthermore, ectopic over-expression of ENPP1 protein in HLA-A2+ tumor cells did not activate TCR-Ts. *In silico* analysis showed that the ENPP1_436_ peptide affinity for HLA-A0201 was ranked 40 times lower than AFP_158_ and the chance of ENPP1_436_ peptide being processed and presented by HLA-A0201 was 100 times less likely than AFP_158_. In contrast, the two off-targets (Titin and MAGE-A3) that did cause severe toxicity in previous trials have the same or higher MHC-binding affinity and the same or higher chance of being processed and presented. In conclusion, our data shows that TCR-Ts can be activated by off-target ENPP1_436_ peptide. But, compared to target AFP_158_, it requires at least 250 times more ENPP1_436_ to achieve the same level of activation. Importantly, ENPP1_436_ peptide in human cells is not processed and presented to a sufficient level to activate the AFP_158_-specific TCR-Ts. Thus, these TCR-Ts, especially the TCR1-Ts and TCR2-Ts, will unlikely cause significant off-target toxicity.

## Introduction

With 840,000 new diagnoses and 781,000 deaths annually, liver cancer is the 6th most common cancer, and the 3rd most common cause of cancer deaths due to the lack of effective treatment (1). The majority of liver cancer is hepatocellular carcinoma (HCC). Recently, several immunotherapies are being developed for HCC (2). The PD1 blockade has significantly increased the overall response rate (3). But its effect may depend on the presence of tumor reactive T cells (4), which are not always present in most HCC lesions. Redirecting autologous T cells with tumor antigen-specific T cell receptor (TCR) genes will provide the tumor-specific T cells, and thus has a great potential for cancer immunotherapy (5). The feasibility of TCR gene transfer to render T cell specificity was published in 1986 (6). And the first evidence that TCR gene engineered T cells (TCR-Ts) generated antitumor effect in treating human cancers was reported 20 years later in 2006 (7). Since then, a number of human tumor antigen specific TCRs derived from both mouse and human sources, including the TCRs specific for MART1 (human TCR) (8), GP100 (mouse TCR) (8), CEA (mouse TCR) (9), NY-ESO1 (human TCR) (10, 11), and MAGE-A3 (mouse and human TCR) (12, 13), have been tested in clinical trials. Adoptive transfer of TCR-Ts has generated significant antitumor effect in several cancers (14). The clinical trial data from NY-ESO1 specific TCR-Ts in treating melanoma, multiple myeloma, and synovial carcinoma is very promising with great safety profile (10, 11). Recently, we (15) and others (16) identified the human alpha fetoprotein (AFP)- specific TCR genes from mouse and human and showed that human T cells genetically modified with the AFP-specific TCRs could effectively kill HCC tumor cells and eliminated HCC xenografts in immune compromised NSG mice (15), demonstrating the potential of the TCR-Ts for HCC immunotherapy.

While the antitumor potency of TCR-Ts is evident, many of the TCR-Ts trials, such as those against CEA, GP100, MART, and MAGE-A3 antigens, also showed significant toxicity including patient death. The toxicity of TCR-Ts can come from three aspects: (1) the on-target/off tumor toxicity due to the low level expression of shared-tumor antigen in normal tissues (8, 9); (2) the off-target toxicity due to the TCR’s promiscuous recognition of unrelated epitopes derived from normal proteins (12, 13, 17); and (3) the alloreactivity of TCR-Ts recognizing different HLA presented random peptides. All three aspects of the TCR-T’s toxicity must be properly evaluated prior to conducting clinical trials. As investigations of TCR-Ts in animal models offers little value in evaluating their toxicity in human, an *in vitro* preclinical toxicity study strategy was proposed to assess the TCR-T’s risk (18). Ideally, tumor-specific or relatively tumor-specific antigens should be selected as the TCR-T’s target to reduce on-target/off-tumor reactivity. However, even with highly tumor-specific targets, the off-target cross-reactivity of TCR-Ts in recognition of MHC-peptide complex may still cause severe toxicity.

In this report and the accompanying study, we determined the optimal TCRs out of the 7 AFP_158_-specific TCRs based on their preclinical antitumor efficacy and toxicities. The selection of optimal TCR-Ts for HCC immunotherapy and the on-target/off-tumor toxicity and alloreactivity of the AFP_148_-specific TCR-T’s were reported in the accompanying paper (Luo et al). In this study, we investigated the off-target cross reactivity of 3 potent TCR-Ts by using X-scan. We found that TCR3-Ts could be cross-activated by 2 synthetic peptides, the ENPP1_436_ and RCL1_215_, while the TCR1-Ts and TCR2-Ts were activated by only ENPP1_436_. The EC_50_ of ENPP1_436_ peptide for activating AFP_148_-specific TCR-Ts was 17–33 times higher than the EC_50_ of AFP_158_. And it required 250–400 times more of ENPP1_436_ and 1000 times of RCL1_215_ peptide to achieve the same level of TCR-T activation as AFP_158_ peptide. Importantly, the HLA-A020 + ENPP1 + human cells do not activate TCR1-Ts and TCR2-Ts. In addition, *in silico* analysis showed that the ENPP1_436_ peptide’s MHC binding affinity and its chance of being processed and presented by HLA-A0201 were significantly lower than that of AFP_158_. In contrast, the two off-targets (Titin and MAGE-A3) that indeed caused severe toxicity in previous trials had the same MHC binding affinity and the same or higher chance of being processed and presented by host cells. Altogether, we conclude that the AFP_158_-specific TCR-Ts, especially TCR1-Ts and TCR2-Ts, will not cause significant off-target toxicity.

## Materials and Methods

### Cells

The cell lines of TAP^–/–^ T2 (19), HepG2, and HEK293 were purchased from American Type Culture Collection (ATCC, Manassas, VA, United States). Breast cancer cell lines of MCF7, MDA-MB231, and brain tumor U87MG cells were purchased from ATCC. MDA-MB231-Luc cells were derived from MDA-MB231 by transfecting with luciferase gene and kindly provided by Dr. Hasan Korkaya of Georgia Cancer Center. The cells were cultured in standard DMEM or RPMI1640 media for no more than 8–10 passages to maintain their authenticity. Mycoplasma test was conducted according to manufacturer instructions (Thermo Fisher, MA, United States). Primary normal adult human hepatocytes were purchased from Lonza (Walkersville, MD, United States) and Novabiosis (Research Triangle Park, NC, United States).

### Peptides

Peptides were synthesized by GenScript (Piscataway, NJ, United States) and Chinapeptides (Shanghai, China) to a purity of >95%. The stock peptides are dissolved in DMSO at 5 mg/ml and aliquoted and stored at −20°C.

### T Cell Isolation and TCR Transduction

Buffy coat was obtained from local Shepard blood center. PBMCs were harvested by centrifugation on a Lymphoprep Ficoll gradient, diluted to 1 × 10^7^ cells/ml, aliquoted, and frozen. Lentiviral vectors expressing TCRs were prepared by 4 a plasmid co-transfection as previously described (20–22). Total T cells were isolated from PBMC by negative isolation kit (STEMCELL Technologies, Vancouver, BC, Canada) and then transduced by lentiviral vectors at MOI of 20–40 as described (15). Between 12–15 days after transduction, tetramer (NIH Tetramer Core Facility) staining was conducted to measure the percentage of TCR+ T cells. The TCR-Ts were then aliquoted and frozen.

### X-Scan Assay

X-Scan assay was performed as described (23). Briefly, one vial of TCR-Ts were thawed and recovered 2–3 days before use. For each well of the 96 well plate, 15,000 T cells (∼5,000 TCR-Ts) were cultured with 20,000 T2 cells in the presence of 10 ng/ml of X-peptides, which is equal to the EC_90_ of the index AFP_158_ peptide. After co-culture overnight, the media was collected and assayed for IFNγ by ELISA. The absolute amount of IFNγ after X-peptides was then compared to the IFNγ level after AFP_158_ peptide stimulation, and the ratios were calculated and presented.

### Immune Analysis

IFN-γ ELISA kits and antibodies were from Biolegend (San Diego, CA, United States). HLA-A0201/AFP_158_ Tetramer was synthesized by NIH Tetramer Core facility. Antibody staining was done according to each antibody’s instructions. Flow cytometry was done on BD LSRII (San Jose, CA, United States). Data was analyzed using the FCS express software (*De Novo* Software, Pasadena, CA, United States).

### Ectopic Over-Expression of Ectonucleotide Pyrophosphatase/Phosphodiesterase 1 (ENPP1) Protein

MCF7 cells were transduced by ENPP1 gene (Addgene, Watertown, MA, United States) by utilizing Lipofectamine 2000 (Invitrogen, Carlsbad, CA, United States). The expression of ENPP1 was detected by Western blot (WB) analysis (Figure 5A).

### WB Analysis

Protein expression in cell lines was examined by WB analysis. Briefly, cells was homogenized in RIPA lysis buffer containing 25 mmol/L Tris–HCl pH 7.6, 150 mmol/L NaCl, 1% NP-40, 1% sodium deoxycholate, 0.1% SDS and protease inhibitor cocktail (Sigma Aldrich, St. Louis, MO, United States). Protein samples were resolved on 8% SDS polyacrylamide gel. The protein was transferred onto nitrocellulose membrane, which was blocked by using 5% (w/v) non-fat dried milk in Tris–buffered saline containing 25 mM Tris–HCl (pH 7. 4), 130 mM NaCl, 2.7 mM KCl and 0.1% Tween 20. The blot membranes were probed with anti-ENPP1 antibody (Genscript, Piscataway, NJ, United States) and the horseradish peroxidase conjugated anti-IgG secondary antibody (Cell Signaling, Danvers, MA, United States), followed by lightning ECL (PerkinElmer, Waltham, MA, United States).

### *In silico* Analysis

The online software of NetMHC4.0 (24, 25), IEDB (26), and NetCTLpan (27) were used to analyze the ranks of peptide’s MHC binding affinity and peptide’s chance of being processed and presented. The rank of MHC binding affinity by NetMHC4.0 is based on a total of 400,000 random natural peptides in its databank and the rank of peptide’s chance of being processed and presented by HLA-A0201 is based on 200,000 random natural peptides in the NetCTLpan databank.

### Statistics

Statistical analysis was done with Prism software using either ANOVA or student *t-*test.

## Results

**FIGURE 1:**
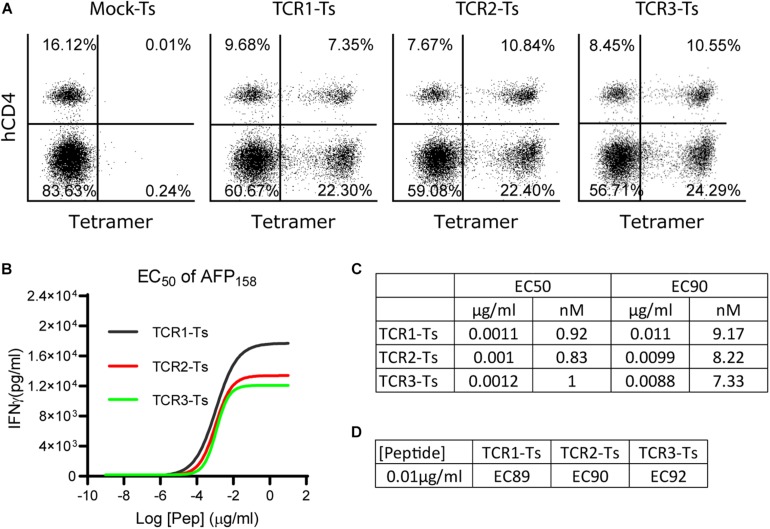
The EC_50_ and EC_90_ of AFP_158_ peptide for activating TCR-Ts. **(A)** TCR-Ts were generated by transducing primary human T cells with TCR genes. Representative dot plots showed the% of TCR + T cells 12–15 days after transduction. **(B,C)** The EC_50_ of AFP158 peptide for activating TCR-Ts were presented. The EC_90_ was calculated using Prism software. **(D)** The EC_XX_ value corresponding to the 10 ng/ml of AFP_158_ peptide for activating different TCR-Ts was calculated using Prism software. The experiments were repeated three times with similar data.

We have identified 7 unique AFP_158_-specific TCR sequences. A functional screening (Refer to Figure 1 of the accompanying paper, Luo et al) identified that human T cells engineered with TCR1, 2, and 3 genes (TCR1- TCR2-, and TCR3-Ts) generated strong cytotoxicity and produced high level of cytokines, which were consistent with our previous report (15). Thus, in the study of off-target cross-reactivity, only the TCR1-, 2-, and 3-Ts were used.

### The EC_50_ and EC_90_ of Target AFP_158_ and the Peptide Concentration for X-Scan

To comprehensively study the TCR-T’s off-target reactivity, X-scan assay was recently developed (23). To properly conduct an X-scan, we first need to decide the right peptide concentration for the assay. To this end, the EC_50_ of index AFP_158_ peptide for activating AFP_158_-specific TCR-Ts was determined. TCR-Ts were prepared by transducing primary human T cells with lentiviral vectors. Approximately 30–35% of T cells were stained positive by HLA-A0201/AFP_158_ tetramer, and 2/3 of the TCR + T cells were CD8 (Figure 1A). TCR-Ts were co-cultured with T2 cells in the presence of different concentrations of AFP_158_ peptide. The IFNγ in the media was measured 20 h later. Using this approach, we determined the EC_50_ of AFP_158_ peptide for activating the TCR1-Ts, TCR2-Ts, and TCR3-Ts were 1.1 ng/ml (0.92 nM), 1 ng/ml (0.83 nM), and 1.2 ng/ml (1.0 nM), respectively (Figures 1B,C). This AFP_158_ EC_50_ value for activating our TCR-Ts is similar to the EC_50_ of AFP_158_ peptide for activating human TCR-Ts recently reported by Docta et al. (16), but is slightly higher than several EC_50_ previously reported for other affinity-enhanced TCR-Ts. For example, the EC_50_ of cognate peptide for activating the MAGE-A10 specific TCR-Ts was 0.3–0.5 nM (23), and the EC_50_ of NY-ESO1_157_ peptide for activating cognate TCR-Ts was around 0.2 nM (28). In contrast, the EC_50_ of cognate peptide for activating the MAGE-C2 specific TCR6-T cells was 3.3 nM (18). Next, based on the EC_50_, the EC_90_ of AFP_158_ peptide was calculated as 10 ng/ml (Figures 1C,D). As recently reported by Border et al. (23), the EC_90_ of AFP_158_ was selected as the peptide concentration for conducting X-scan assay.

### X-Scan Assay Determines the Peptide Motifs That Are Potentially Recognized by TCR-Ts

**FIGURE 2:**
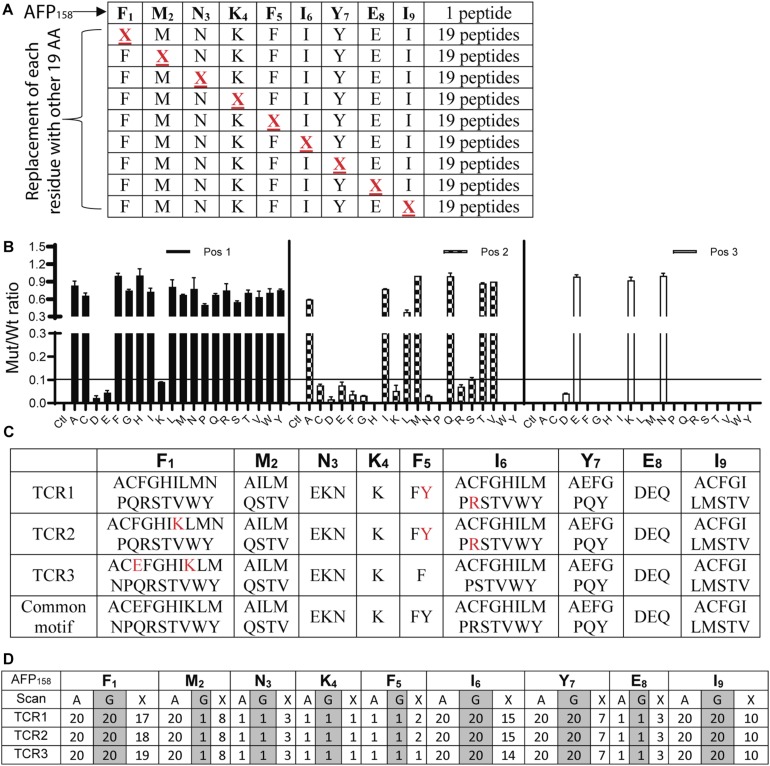
Identification of peptide motifs that can be recognized by TCR-Ts. **(A)** The scheme of X-Peptides. “X” in each position represents any of the other 19 amino acids. **(B)** A representative data (position 1–3 of TCR1-T) of X-Scan assay showed the response ratio of X-peptide vs. AFP_158_ peptide in stimulating TCR-Ts to produce IFNγ. The Mean ± SD from 3 wells was shown. The X-scan assay for each peptide was repeated 3–5 times and consistent observation was obtained. **(C)** X-Scan identified the peptide motifs potentially recognized by TCR-Ts. The colored letters indicate the difference among 3 TCR-Ts. A common motif that cover all 3 TCR-Ts is also shown. **(D)** Comparison of A-, G-, and X-Scan to reveal the number of the tolerant amino acids at each position AFP_158_ epitope.

To conduct X-scan, each amino acid residue of the index AFP_158_ peptide was replaced with every other possible amino acids to create a library of X-peptide (Figure 2A). A total of 171 X-peptides and the original index AFP_158_ peptide were used to stimulate TCR-Ts. An amino acid substitution in the X-peptide was defined as tolerant if the TCR-T response stimulated by this peptide was >10% of original AFP_158_ peptide. X-scan allowed us to identify all tolerant amino acid replacements in each position (Figure 2B). The complete X-scan data was shown in Supplementary Figure S1. Based on the X-scan data, a peptide motif for each TCR-T was generated (Figure 2C). We also conducted the Alanine (A)-scan and Glycine (G)-scan according to previous report (17) (replacing each residue in the target epitope with Alanine or Glycine) (Supplementary Figure S2). While the data from A- or G- Scan can only indicate either 1 (intolerant) or 20 (tolerant), the motif obtained from the X-scan reveals more precise amino acid replacements (Figures 2C,D).

### AFP_158_-Specific TCR-Ts Can Be Cross-Activated by the ENPP1_436_ Peptide

**FIGURE 3:**
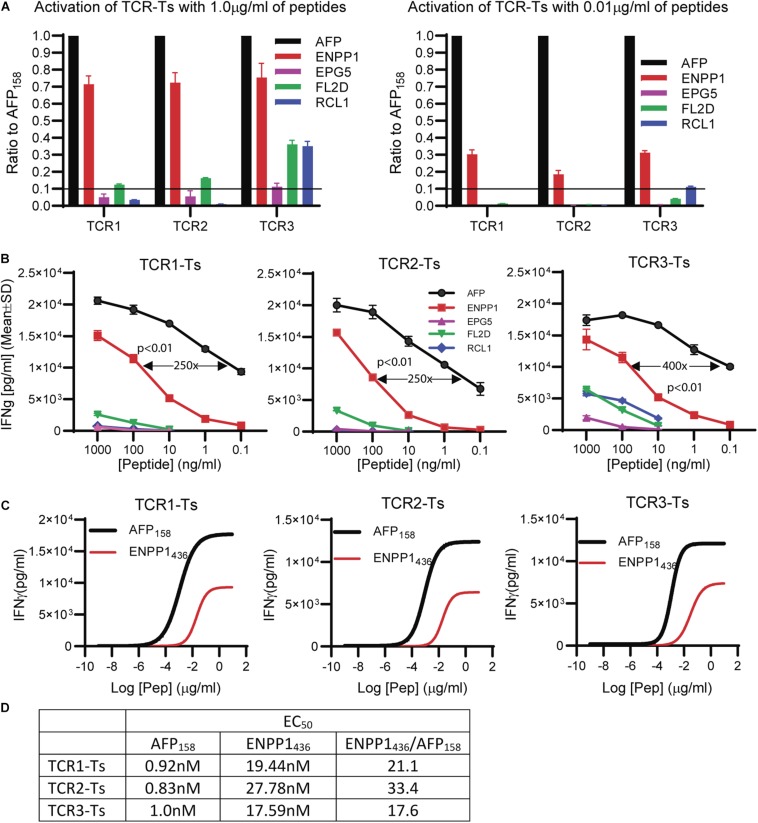
Synthetic off-target peptides cross-activate TCR-Ts. **(A)** The activation of TCR-Ts to produce IFNγ by 5 peptides at high (1 μg/ml) and low (0.01 μg/ml) concentrations is presented. The data shown was the response ratios of off-target peptides vs. AFP_158_. **(B)** Shown is the dose-dependent production of IFNγ by TCR-Ts after stimulation with different peptides. ANOVA was used for statistical analysis. **(C,D)** The EC_50_ of AFP_158_ and ENPP1_436_ was measured and compared.

The peptide recognition motifs of TCR1, 2 and 3 are slightly different (Figure 2C). To make sure that we would not miss any potential reactive peptides, we used a common peptide motif (Figure 2C) that cover all 3 TCRs to search the SwissProtein databank using the ScanProsite program (29). A total of 93 peptides with the potential capability of activating the TCR-Ts were identified and synthesized (Supplementary Table S1). In the 1st experiment, we used high peptide concentration (1 μg/ml) in the stimulation assay to catch all potential peptides that may activate TCR-Ts. By using 10% of the AFP_158_ response as cut-off, we found that TCR1-Ts and TCR2-Ts could be cross-activated by ENPP1_436_ and FL2D_189_ peptide. TCR3-Ts could be cross-activated by four peptides, the ENPP1_436_, FL2D_189_, EPG5_1033_, and RCL1_215_ peptides (Figure 3A and Supplementary Figure S3). But, at a lower peptide concentration (10 ng/ml), TCR1-T and TCR2-T could be cross-activated by ENPP1_436_ only, while TCR3-Ts were cross-activated by both ENPP1_436_ and RCL1_215_ peptides. A peptide titration study showed that it required high concentration (1 μg/ml) of FL2D_189_, EPG5_1033_, and RCL1_215_ to activate TCR-Ts. At 10 ng/ml, the RCL1_215_ could weakly cross-activate TCR3-Ts. The data also showed that it required 10,000 times more RCL1_215_ peptide to achieve the same level of activation as AFP_158_. Even for the more reactive ENPP1_436_, it would require 2 log more (250–400 times) of peptide to generate the same level of response as AFP_158_ (Figure 3B). Furthermore, the EC_50_ of ENPP1_436_ for cross-activating the AFP_158_-specific TCR-Ts were 17–33 times higher than AFP_158_ (Figures 3C,D).

### ENPP1 Expressing Cells Do Not Activate TCR-Ts

**FIGURE 4:**
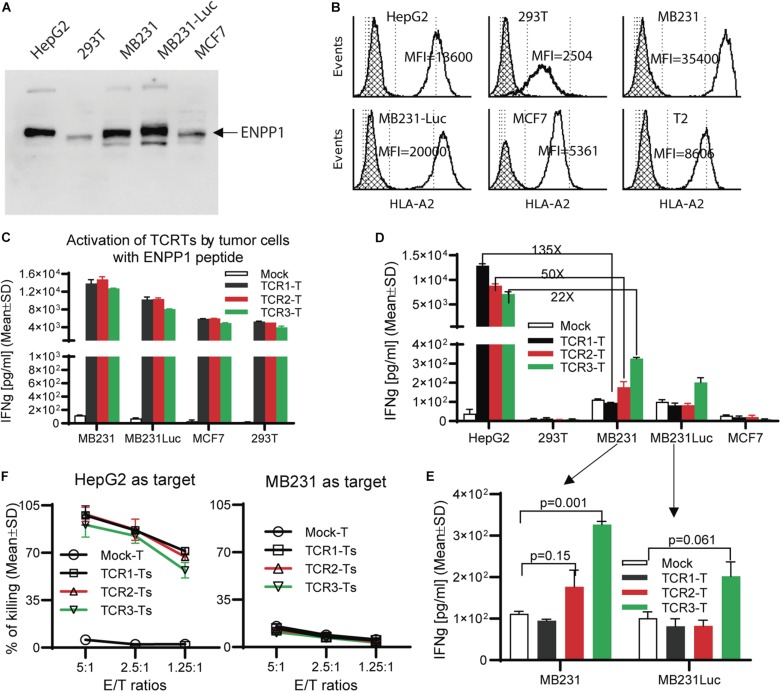
TCR-Ts do not recognize and kill ENPP1 + tumor cells. **(A)** Western Blot analysis showed ENPP1 expression in different cell lines. MB231-Luc was derived from MDA231 by transfecting Luciferase gene. **(B)** Flow cytometry data demonstrated the HLA-A2 expression level on the surface of different cell lines by anti-HLA-A2 antibody staining. The hatched lines indicate the isotype staining controls. **(C)** ELISA assay showed the level of IFNγ production by TCR-Ts after stimulation with indicated cell lines in the presence of 1 μg/ml ENPP1 peptide. **(D,E)**. The activation of TCR-Ts by different cell lines in the absence of ENPP1 peptides was presented. Only the TCR3-Ts were weakly activated, while TCR1-Ts and TCR2-Ts could not be activated by ENPP1 + MB231 and MCF7 cells. **(F)** LDH assay showed that TCR-Ts do not kill the ENPP1 + MB231 cells. HepG2 cells were used as positive control. Student *t-*test was used for statistical analysis. This experiment was repeated 4 times with similar data.

A number of human tissues express ENPP1^1^. Thus, we studied whether the ENPP1 expressing cells could cross-activate AFP_158_-specific TCR-Ts. To this end, we detected ENPP1 in several cell lines and found out that MB231 and MCF7 are ENPP1 + (Figure 4A) and HLA-A2 +. The MDA-MB231 cells and MB231-luc (derived from MB231 and expressing luciferase) have a very high level of HLA-A2 (10 times more A2 than 293T cells) (Figure 4B). Consistent with the highest level of HLA-A2, MB231 pulsed with ENPP1 peptide stimulated the highest activation of TCR-Ts (Figure 4C). However, even though the MB231 and MB231-luc express high level of ENPP1 and HLA-A2, they did not activate TCR-Ts (Figure 4D). A detail analysis revealed that, while TCR1-Ts and TCR2-Ts did not produce more IFNγ than Mock-T cells after MB231 stimulation, the TCR3-Ts produced slightly more IFNγ (∼300 pg/ml by TCR3-Ts vs. 100 pg/ml by Mock-Ts) (Figure 4E). But the IFNγ level produced by TCR3-Ts after MB231 stimulation was 22x lower than that after HepG2 stimulation (Figures 4D,E). The *in vitro* CTL assay showed that all 3 TCR-Ts were unable to kill MB231 tumor cells (Figure 4F). To further test whether TCR-Ts could be activated by ENPP1 expressing cells, we then used U87MG brain tumor cells, which express higher level of ENPP1 than MB231 and similar level of HLA-A2. Again, all TCR-Ts were not cross-activated by and did not kill the U87MG tumor cells (Supplementary Figure S4). In addition, normal primary hepatocytes also express ENPP1 (Supplementary Figure S5A). The hepatocytes from 2 donors were also HLA-A2+ (refer to Figure 3C of the accompanied paper, Luo et al). The data showed that hepatocytes did not activate TCR-Ts (Supplementary Figure S5B), consistent with our previous report (15).

### Ectopic Over-Expression of ENPP1 in MCF7 Did Not Activate TCR-Ts

**FIGURE 5:**
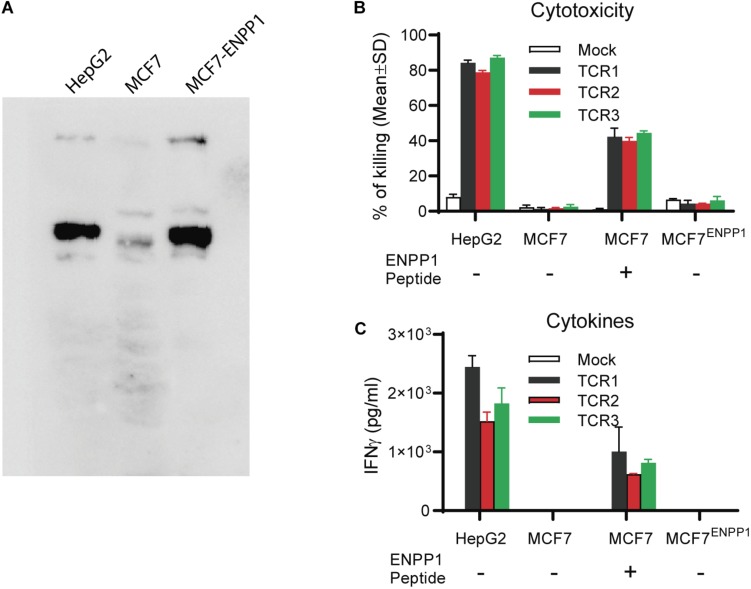
Tumor cells overexpressing ENPP1 do not activate TCRTs. **(A)** Western Blot analysis showed the ectopic overexpression of ENPP1 in MCF7 cells after transient gene transfer. **(B)** LDH assay showed that TCR-Ts did not kill the MCF7 cells that overexpress ENPP1 protein. **(C)** ELISA assay indicated that overexpression of ENPP1 in MCF7 did not activate TCR-Ts to produce IFNγ.

In this experiment, we studied whether ectopic over-expression of ENPP1 in MCF7 tumor cells could cross-reactivate TCR-Ts. We found that while all 3 TCR-Ts could be activated by and kill the MCF7 pulsed with ENPP1_436_ peptide, they were not activated by and did not kill the ENPP1 overexpressing MCF7 tumor cells (Figures 5A–C). In conclusion, all the data indicates that it is unlikely that the ENPP1 expressing cells in the patients will cross-activate AFP_158_-specific TCR-Ts.

### *In silico* Analyses May Predict TCR-T’s Off-Target Reactivity

Here we studied whether *in silico* analysis would help predict TCR-T’s off-target reactivity. First, we used the NetMHC4.0 program to rank the 93 peptide’s MHC binding affinity based on databank of 400,000 random natural peptides. The results were then correlated to the experimental data of peptide’s TCR-T activation. The data was summarized in Table 1 and Supplementary Table S1. According to the default criteria of 0.5% set by the software, only AFP_158_ (rank: 0.01%) and ENPP1_436_ (rank: 0.4%) peptides are strong binders of HLA-A0201. Consistent with *in silico* analysis, the experimental data showed that these two peptides could activate all 3 TCR-Ts at physiological concentration (10 ng/ml). In contrast, RCL1_215_ (rank: 0.8%) is a weak binder. The experimental data showed that RCL1_215_ cross-activated only TCR3-Ts (Figure 3A). FL2D_189_ (rank: 17%) is a non-binder. It weakly cross-activated TCR-Ts only at high concentration. The EPG_1033_ peptide (rank: 29%) is also a non-binder. It activated only TCR3-Ts at high concentration. The MHC binding affinity of peptides was also analyzed by IEDB software and its rank was similar to NetMHC4.0 (Table 1). Secondly, we used the NetCTLpan program to rank a peptide’s chance of being processed and presented by MHC molecule. Based on the data of 200,000 random natural peptides, RCL1_215_ and ENPP1_436_ peptides are ranked at 0.8% and 1% (Table 1), which means they are the top 1600 and 2000 peptide of being processed and presented, respectively. In contrast, the AFP_158_ is ranked at 0.01% (Table 1), which means it is the top 20 out of 200,000 peptides that are processed and presented. Thus, AFP_158_ has much higher probability of being processed and presented. In conclusion, the *in silico* analysis data of peptide’s MHC binding affinity and their chance of being processed and presented correlate well with the experimental data of cross-reactivity.

**TABLE 1:** Correlation of the *in silico* analysis data with experimental data of cross-reactivity.

		MHC binding affinity			Ranks (%) of being presented	Experimental data	
Epitope	Peptide Sq	NetMHC 4.0		IEDB	NetCTLpan	[Peptide] (μg/ml)	
		Affinity (nM)	Rank (%)	Rank (%)		1.0	0.01
AFP_158_	FMNKFIYEI	3.2	0.01	0.2	0.01	++++	++++
ENPP1_436_	YLNKYLGDV	28.67	0.4	0.6	1	+++	+
RCL1_215_	ILNKFIPDI	64.06	0.8	2	0.8	−/−/++	−/−/+
FL2D1_89_	LQKKYSEEL	11170.64	17	22	6	+/+/++	−/−/−
EPG5_1033_	SIEKFCAEG	20847.93	29	37	50	−/−/+	−/−/−

*The peptide’s HLA-A2 binding affinity is analyzed by NetMHC4.0 and IEDB software. The likelihood (rank) of the peptides being processed and presented by MHC molecules is analyzed by NetCTLpan program. The lower the number, the higher the chance of that peptide being processed and presented. The rank of NetMHC4.0 binding affinity is based on 400,000 random natural peptides. The ranks of the peptide being processed and presented is based on 200,000 random natural peptides. The wet laboratory experimental data of cross reactivity is shown in the last column. In the last column where the reactivity is different among TCR1, 2, and 3, their reactivity is indicated as TCR1/TCR2/TCR3.*

In addition, we did retrospective *in silico* analysis of the peptides that were known to cause severe toxicity in previous trials. It was reported that the HLA-A01/MAGE-A3 specific TCR-Ts (17) and HLA-A0201/MAGE-A3 specific TCR-Ts (30, 31) caused severe cardiac and neurological toxicity by cross-reacting with off-target peptides of Titin in the heart and MAGE-A12 in the brain, respectively. We compared the peptides of MAGE-A3 vs. Titin and MAGE-A3 vs. MAGE-A12 regarding to their MHC binding affinity and chance of being processed and presented by *in silico* analysis. The MAGE-A3 and Titin peptides have the same ranking of binding to HLA-A01 (0.01% vs. 0.01% by NetMHC4.0; 0.12% vs. 0.17% by IEDB software) (Table 2). Both epitopes also have the same chance (0.05%) of being processed and presented (Table 2). In the second example, MAGE-A12 epitope (rank 0.01%) has an even higher affinity than index MAGE-A3 peptide (rank 0.03%) for binding HLA-A0201. The MAGE-A12 epitope also has higher chance of being processed and presented by HLA-A0201 than the index MAGE-A3 peptide (0.2% vs. 0.01%) (Table 2). Thus, in both cases that TCR-Ts caused severe toxicity, *in silico* analysis showed that the off-target peptide’s MHC binding affinity and chance of being processed and presented were at least at the same level as the intended index epitopes.

**TABLE 2:** *In silico* analysis of two previous TCR-Ts that showed severe toxicity in clinical trials.

	Antigen	Epitope	NetMHC4.0		IEDB Rank (%)	NetCTL pan	Cross-Reactivity
			Affinity (nM)	Ranks (%)			
A0101/MAGE-A3	MAGE-A3	EVDPIGHLY	11.43	0.01	0.12	0.05	+++
	Titin	ESDPIVAQY	8.07	0.01	0.17	0.05	++
A0201/MAGE-A3	MAGE-A3	KVAELVHFL	16.05	0.25	0.9	0.2	+++
	MAGE-A12	KMAELVHFL	3.28	0.01	0.2	0.01	++++
	MAGE-A2	KMVELVHFL	4.61	0.03	0.3	0.05	+
	MAGE-A4	KVDELAHFL	60.75	0.70	1.9	0.8	−
	MAGE-A6	KVAKLVHFL	109.34	1.10	2.2	1.5	+
	MAGE-A1	KVADLVGFL	165.35	1.50	2.8	1.5	−
	MAGE-A8	KAVELVRFL	2082.94	6.00	7.6	3	−

*The cross-reactivity in vitro experimental data are taken from the references of 17 and 31. The shade data indicate the cross reactivity that cause severe in vivo toxicity. The rank of NetMHC4.0 binding affinity is based on 400,000 random natural peptides. The ranks of the peptide being processed and presented by NetCTL program is based on 200,000 random natural peptides.*

## Discussion

### The Reliability of X-Scan

T cell activation requires their TCR to specifically bind to cognate MHC/peptide complex. However, the TCR’s recognition of MHC/peptide complex is degenerate (32), which may cause lethal off-target cross-reactivity (12, 30). X-scan is a comprehensive approach to study TCR-T’s off-target reactivity (16, 23). But its reliability of X-scan may depend on the X-peptide concentration used for the assay. Conducting the assay with too high concentration of X-peptide will unnecessarily find many peptides that cross-activate TCR-Ts, but in reality will never do so because of their lower physiological level. On the other hand, too low of concentration will miss out the peptides that may do cause cross reactivity. In our study, we found that the EC_50_ of AFP_158_ peptide for activating AFP_158_-specific TCR-Ts was ∼1 nM. According to previous study (33), T2 cells pulsed with 1 nM of gp100 peptide and WT-1 peptides yielded 12–47 copies of MHC/peptide complex on cell surface. This number of MHC/peptide complex is similar to the naturally presented gp100 epitopes on the melanoma Mel526 and Mel624 cells (9–68 copies) (33) and the naturally processed MHC/NY-ESO1 peptide complex on tumor cells (34). Therefore, 1 nM is the relevant concentration for X-scan that generates physiological level of MHC/peptide complex on cell surface. Thus, we agree that the EC_90_ of index peptide used in a recent study (23) is the right concentration for X-scan assay in order to make sure that no potential peptides will be missed out.

The reliability of X-scan may also be affected by the fact that the X-peptide contains only one amino acid residue replacement while all other amino acid residues remain original. It does not consider the effect of other amino acid residues in the epitope that may also affect its capability of activating TCR-Ts. Thus, it is possible that peptides with more than one amino acids being simultaneously changed may not follow the rule concluded from X-scan assay. For example, the Y replacement at position 5 of AFP_158_ was intolerant for TCR3-T activation (even though it is tolerant for TCR1-T and TCR2-T) (Supplementary Figure S1). However, contradictory to this rule, the ENPP1_436_ peptide is capable of activating TCR3-Ts (tolerant) even it has Y at position 5. Compared to AFP_158_ (FMNKFIYEI), the ENPP1_436_ peptide (YLNKYLGDV) has Y at position 5, but also has other 7 amino acid replaced. Those additional amino acid changes may work together to enhance the peptide’s activation of TCR-Ts. To minimize the chance of missing any potential reactive peptides, for the three positions (E and K at Position 1; Y at Position 5; R at Position 6) in the peptide motifs that are different among the 3 TCR-Ts, we used a common peptide motif to cover all potential amino acids to search the protein databank. In this case, as long as one TCR-T is tolerant for one particular amino acid replacement, it will be included in the common motif.

### *In silico* Analysis of Peptide’s MHC Binding Affinity Helps Predict TCR-T’s Off-Target Toxicity

The X-scan strategy may generate a long list of potential off-target peptides. In this study, 93 peptides were identified by X-scan that may potentially cross-activate AFP_158_-specific TCR-Ts. But, the experimental data showed that the peptides indeed cross-activated TCR-Ts at physiological concentration were within the top 1% of their MHC binding affinity ranked by NetMHC4.0 analysis (Supplementary Table S1). The default cut-offs of strong and weaker binder are 0.5 and 2%, respectively. Although more experimental data will be needed, it is reasonable to assume that, at physiological concentration, only the top 2% of peptides ranked by NetMHC4.0 have the potential to truly cross-activate TCR-Ts. Thus, the programs of calculating peptide’s MHC binding affinity, such as NetMHC4.0 and IEDB, may help shorten the peptide list so that wet laboratory experiments will not be too costly.

Secondly, the computer program of NetCTLpan (27), which integrates peptide’s proteasomal C terminal cleavage, TAP transport efficiency, and MHC binding affinity, may help predict the probability of a particular peptide being processed and presented by MHC. The NetCTLpan analysis showed that the two off-target peptides (Titin and MAGE-A12) that indeed cause severe toxicity in human trials had similar or higher chances of being processed and presented as the intended target of MAGE-A3 (Table 2). In contrast, the NetCTLpan ranks that the chance of ENPP1_436_ peptide being processed and presented by HLA-A0201 is 100 times lower than the target epitope AFP_158_ (top 1% vs. top 0.01% in Table 1). Thus, the data of *in silico* analysis is in agreement that the ENPP1_436_ peptide is not efficiently processed and presented.

Thirdly, will TCR bind the index and cross-reactive peptides with similar strength? The recent software to predict TCR and MHC/peptide interaction is highly immature (35). However, this may not be necessary as the ultimate test is the wet experiments. According to previous study (33), as low as 0.1 nM of peptides can stimulate TCR-T to produce IFNγ that can be detected, but it requires 1 nM peptides to generate sufficient level of MHC/Peptide complex on cell surface to be detected by microscope. And, it needs 10 nM of peptides to generate measurable MHC-peptide complex by flow cytometry. Thus, the TCR-T functional activation by measuring the IFNγ level is the most sensitive way of finding potential off-target peptides.

### TCR-T’s Cross-Reactivity to Synthetic ENPP1_436_ Peptide Unlikely Causes Severe Off-Target Toxicity

The cross-activation of AFP_158_-specific TCR-Ts by synthetic ENPP1_436_ peptide raises the concern that AFP_158_-specific TCR-Ts may cause off-target toxicity. However, our experimental data indicates such off-target toxicity is highly unlikely. 1st, the cross-activation of AFP_158_-specific TCR-Ts by ENPP1_436_ peptide is significantly weaker than AFP_158_ peptide. The EC_50_ of ENPP1_436_ peptide for activating the TCR-Ts is 17–33 time higher than AFP_158_ peptide. It requires 250–400 times more of ENPP1_436_ peptide to achieve the same level of activation as AFP_158_ peptide. The significant weak activation of TCR-Ts by ENPP1_436_ peptide is further supported by *in silico* analysis showing that the MHC binding affinity of ENPP1_436_ peptide is ranked 40 times lower than AFP_158_ peptide. Thus, there is a wide concentration window to distinct the intended target from off-target activation. 2nd, the chance of ENPP1_436_ peptide being processed and presented is low. The activation assay of using peptide pulsed T2 cells to stimulate TCR-Ts omits the process of antigen processing which including protein degradation and TAP transport and MHC loading (36). Not all so-called epitopes identified by synthetic peptides can be truly processed and presented by cells (37, 38). In fact, the process of antigen processing and presentation is rather very inefficient, estimating only 1/10,000 peptides being processed and presented (39). The incapability of ENPP1+ MB231, MCF7, U87MG, and normal hepatocytes to activate TCR-Ts is in agreement with the computer prediction that ENPP1_436_ epitope is unlikely being processed and presented by HLA-A0201 to the level that is sufficient to activate TCR-Ts. Thus, we have strong evidence that the AFP_158_-specific TCR-Ts will unlikely cause severe off-target toxicity. That being said, the concern of causing off-target toxicity cannot be completely excluded. Further safety switches such as incorporation of suicide gene (40) and truncated EGFR (41) tag may be added in case of the TCRTs need to be removed.

## Conclusion

In conclusion, our off-target toxicity study showed: (1) The TCR3-T has a broader cross-reactivity than TCR1-T and TCR2-T, highlighting the need of multiple TCRs to find a proper one; (2) The ENPP1_436_ peptide can weakly activate AFP_158_-specific TCR-Ts, but, it requires 250–400 times more of peptide to achieve the same level of activation as AFP_158_ peptide; (3) Importantly, the ENPP1 expressing cells do not activate TCR1-Ts and TCR2-Ts even though they may slightly activate TCR3-Ts; (4) *In silico* analysis show that ENPP1_436_ peptide has a much lower HLA-A0201 binding affinity than AFP_158_ and is less likely of being processed and presented. In contrast, the off-target peptides that indeed cause severe toxicity in previous studies have a similar HLA binding affinity and a similar or even higher chance of being processed and presented than the intended target peptides. Thus, we conclude that it is unlikely that the AFP_158_-specific TCRs will cause significant off-target toxicity.

## Data Availability Statement

The datasets generated for this study are available on request to the corresponding author.

## Author Contributions

LC and YH designed the study, conducted majority of the experiments, summarized the data, and wrote the manuscript. LDC and YP prepared the recombinant DNA, lentiviral vectors, and the TCR-Ts. WZ did the A- and G- Scan experiments. XL, YY, and YJ conducted the Western Blot of ENPP1 in primary hepatocytes and the TCR-T activation by primary normal hepatocytes. All authors agreed the manuscript for publication.

## Conflict of Interest

XL, YY, and YJ are employees of Cellular Biomedicine Group and YH is a part-time consultant for Cellular Biomedicine Group, Inc. The remaining authors declare that the research was conducted in the absence of any commercial or financial relationships that could be construed as a potential conflict of interest.

## Footnotes

1 www.proteinatlas.org

## Abbreviations

AFP: alpha fetoprotein
ENPP1: ectonucleotide pyrophosphatase/phosphodiesterase 1
HCC: hepatocellular carcinoma
RCL1: RNA 3’-terminal phosphate cyclase-like protein
TCR: T cell receptors.
