# Supplementary material for: Preclinical Studies of the Off-Target Reactivity of AFP158-Specific TCR Engineered T Cells
Source: Front Immunol. 2020 Apr 27;11:607. doi: 10.3389/fimmu.2020.00607 (PMC7196607; doi:10.3389/fimmu.2020.00607)
Supplement: Supplementary file 1 [file Data_Sheet_1.PDF]

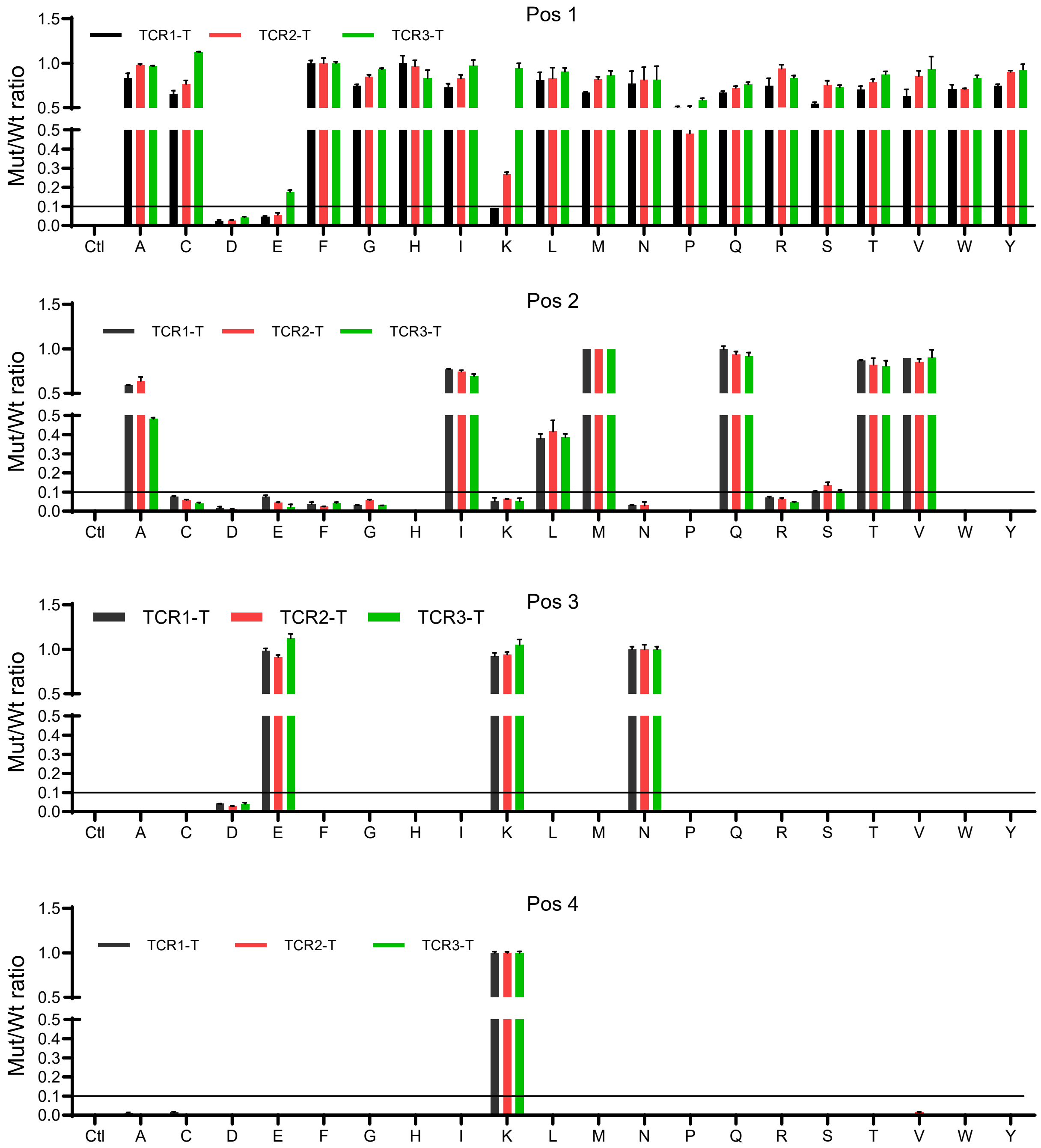

Fig S1A (Position 1-4). X-scan assay identified the tolerable amino acid replacement at each position of AFP_{158} epitope that are able to activate TCR-Ts. X-Peptides with replacement of each amino acid residue with every other 19 amino acids were used to activate TCR-Ts by measuring the IFNg in the co-culture media. The amount of IFNg produced by TCR-Ts after stimulation with mutated epitope was compared to that from AFP_{158} peptide. The ratios was presented.
