# Supplementary material for: Preclinical Studies of the Off-Target Reactivity of AFP158-Specific TCR Engineered T Cells
Source: Front Immunol. 2020 Apr 27;11:607. doi: 10.3389/fimmu.2020.00607 (PMC7196607; doi:10.3389/fimmu.2020.00607)
Supplement: Supplementary file 2 [file Data_Sheet_2.PDF]

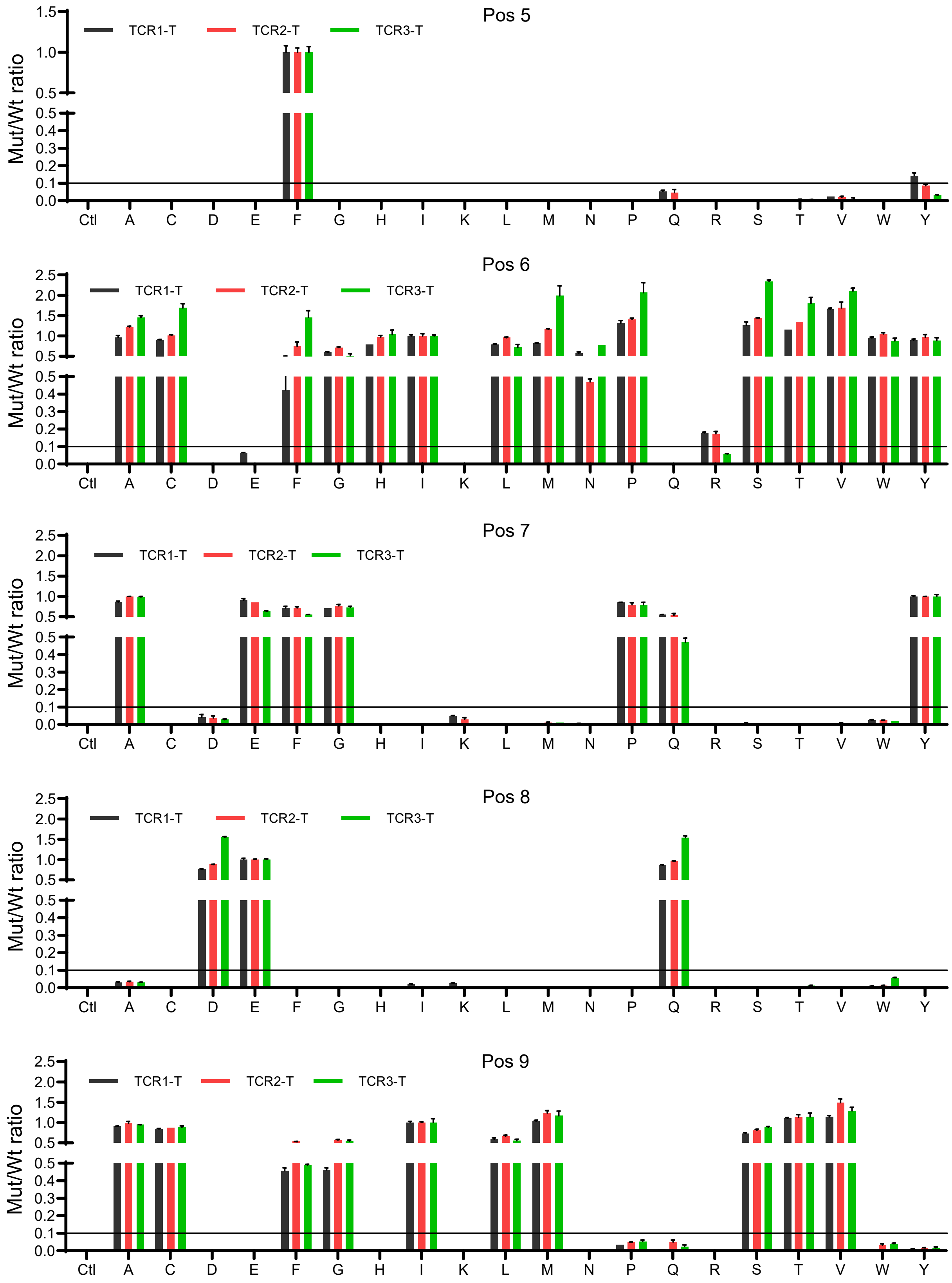

Fig S1B (Position 5-9). X-scan assay identified the tolerable amino acid replacement at each position of AFP158 epitope that are able to activate TCR-Ts.
