# Supplementary material for: Preclinical Studies of the Off-Target Reactivity of AFP158-Specific TCR Engineered T Cells
Source: Front Immunol. 2020 Apr 27;11:607. doi: 10.3389/fimmu.2020.00607 (PMC7196607; doi:10.3389/fimmu.2020.00607)
Supplement: Supplementary file 3 [file Data_Sheet_3.PDF]

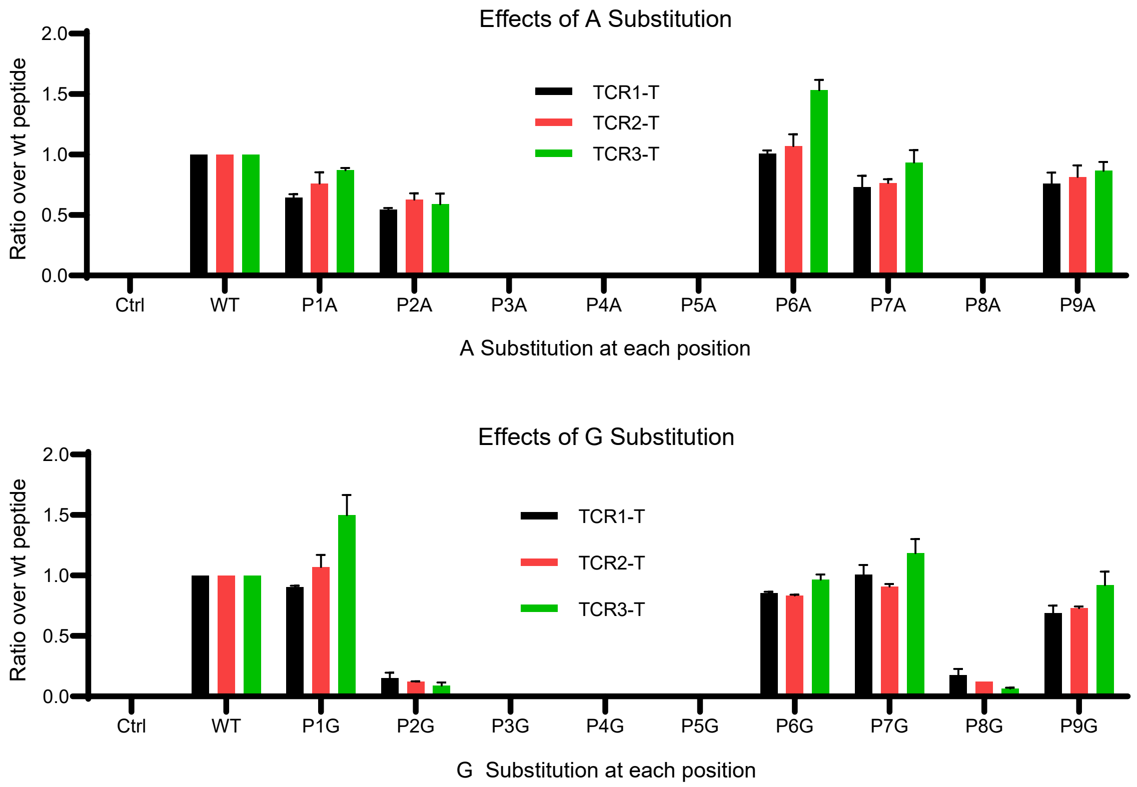

Supplementary Fig S2. A-Scan and G-Scan to identify the critical and tolerable amino acids in the AFP_{158} epitope for their activation o TCR-Ts. The experiment was done similar to Supplementary Fig S1 except the peptides.
