# Supplementary material for: Preclinical Studies of the Off-Target Reactivity of AFP158-Specific TCR Engineered T Cells
Source: Front Immunol. 2020 Apr 27;11:607. doi: 10.3389/fimmu.2020.00607 (PMC7196607; doi:10.3389/fimmu.2020.00607)
Supplement: Supplementary file 4 [file Data_Sheet_4.PDF]

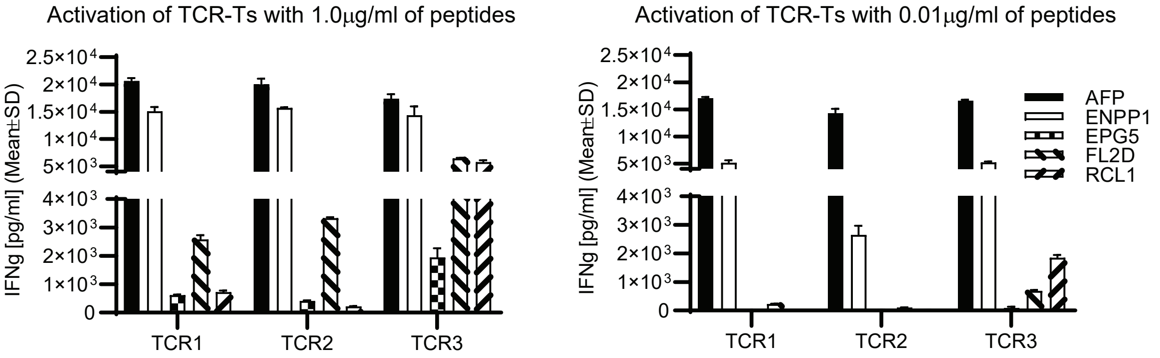

Fig S3. IFNg production (absolute amount) by TCR-Ts after stimulation with high and low concentration of off-target peptides.
