# Supplementary material for: Preclinical Studies of the Off-Target Reactivity of AFP158-Specific TCR Engineered T Cells
Source: Front Immunol. 2020 Apr 27;11:607. doi: 10.3389/fimmu.2020.00607 (PMC7196607; doi:10.3389/fimmu.2020.00607)
Supplement: Supplementary file 5 [file Data_Sheet_5.PDF]

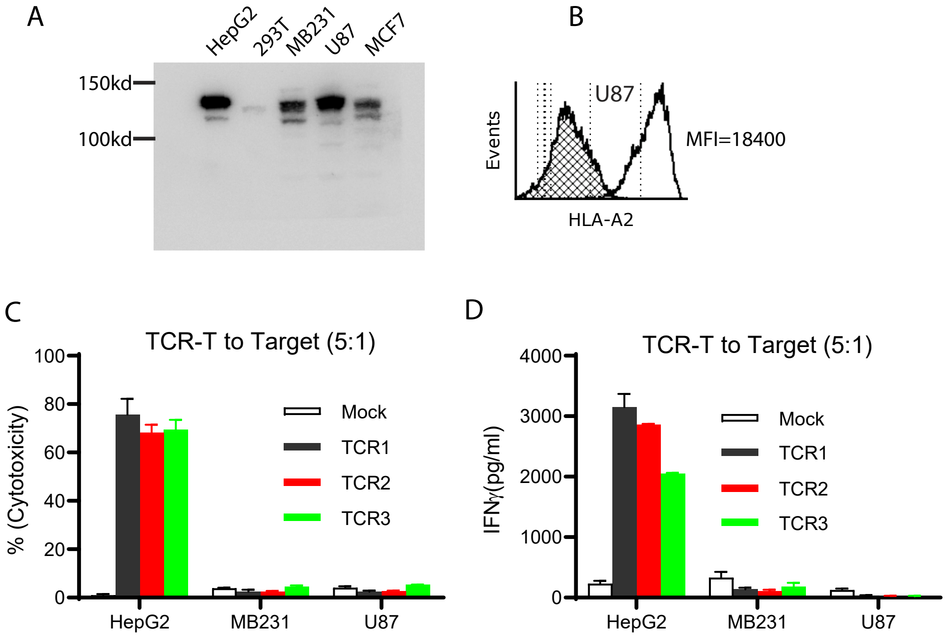

Supplementary Fig S4. Cell line U87 expresses higher level ENPP1 than MB231, but does not activate TCR-Ts, similar to the result of MB231. (A) WB analysis of protein ENPP1 among the indicated cell lines. HepG2 and 293T were 40ug. All other lines were 200ug. (B) the level of HLA-A2 on U87 was shown by FACS. (C) In vitro CTL assay showed that all 3 TCR-Ts did not kill MB231 and U87. (D) ELISA assay indicated that TCR-Ts could not be activated by ENPP1+ MB231 and U87 cells.
