# Supplementary material for: Preclinical Studies of the Off-Target Reactivity of AFP158-Specific TCR Engineered T Cells
Source: Front Immunol. 2020 Apr 27;11:607. doi: 10.3389/fimmu.2020.00607 (PMC7196607; doi:10.3389/fimmu.2020.00607)
Supplement: Supplementary file 6 [file Data_Sheet_6.PDF]

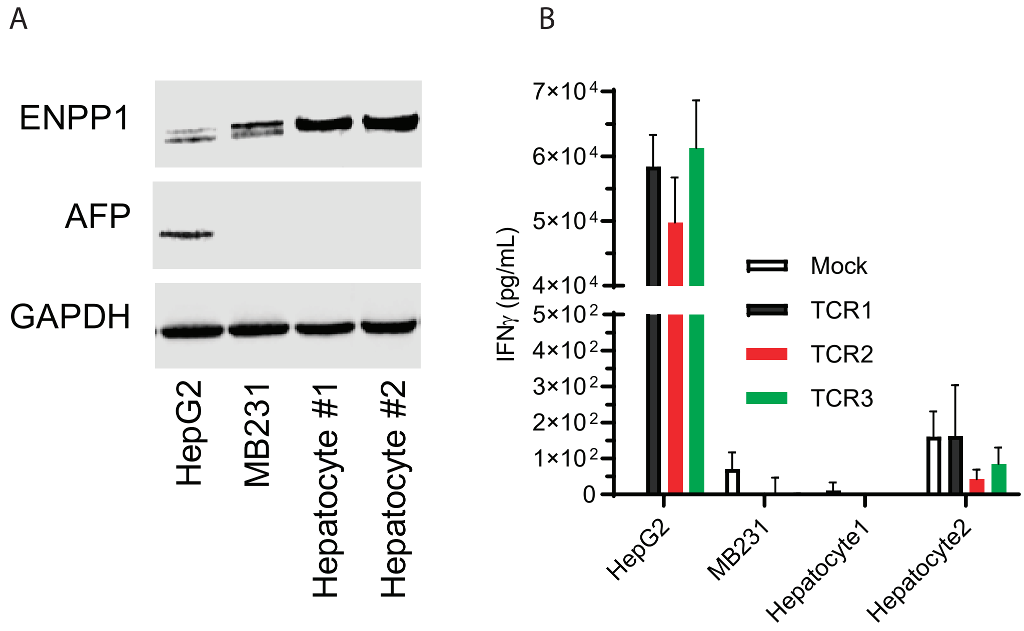

Fig S5. Primary normal hepatocytes do not activate TCR-Ts. (A). WB analysis of AFP and ENPP1 in the primary hepatocytes, HepG2, and MDA-MB231. GAPDH was also detected as control. (B) ELISA assay of IFNg in the coculture media of TCR-Ts and different hepatocytes. Compared to mock-T cells, TCR-Ts were not activated by hepatocytes and MB231 cells, but could be highly activated by HepG2 cells.
