# Supplementary material for: Preclinical Studies of the Off-Target Reactivity of AFP158-Specific TCR Engineered T Cells
Source: Front Immunol. 2020 Apr 27;11:607. doi: 10.3389/fimmu.2020.00607 (PMC7196607; doi:10.3389/fimmu.2020.00607)
Supplement: Supplementary file 7 [file Table_1.pdf]

Supplementary Table S1: Peptides identi ed by searching the Swiss-Prot databank with inclusion of splicing variants using the epitope motif generated by X-Scan and the program of ScanProsite. The affinity ranking is done by NetMHC4.0. SB: strong binder; WB: weak binder. The bolded lines indicate the peptides cross-reacting to TCR-Ts. Yellow shaded line is the index AFP158 peptide.

Peptides	Protein ID.	Protein Name	Epitope	Affinity ranking
1	P55196	AFAD	LAEKFRPDM	48%
2	Q8NB90	AFG2H	LMNKYVGES	16%
3	P02768	ALBU	FLKKYLYEI	0.08% (SB)
4	Q9GZV1	ANKR2	VIEKFLADG	32%
5	Q9HBK9	AS3MT	HMEKYGFQA	7.50%
6	Q13535	ATR	SLEKFVGDA	13%
7	O75309	CAD16	CIEKFSGEV	14%
8	Q9H251	CAD23	NTNKYSFDG	60%
9	P41180	CASR	GIEKFREEA	29%
10	P16152	CBR1	LMNKFVEDT	9%
11	Q8IVW4	CDKL3	FIEKFMPEL	0.6% (WB)
12	Q5SZL2	CE85L	TLEKYLADL	4.50%
13	Q96KN2	CNDP1	RVEKFLFDT	17%
14	Q92564	DCNL4	GMEKFCEDI	9%
15	Q96QD5	DEPD7	IQNKYFGDV	11%
16	P52429	DGKE	GQEKYIPQV	4%
17	Q96EY1	DNJA3	LAKKYHPDT	85%
18	Q8TD57	DYH3	VANKFLEDV	13%
**19**	**P22413**	**ENPP1**	**YLNKYLGDV**	**0.4% (SB)**
**20**	**Q9HCE0**	**EPG5**	**SIEKFCAEG**	**29%**
21	Q5RHP9	ERIC3	MAEKFREEA	31%
22	Q6ZTR7	FA92B	NTEKYFGQF	55%
23	Q9NYQ8	FAT2	NANKYSPEF	50%
**24**	**P02771**	**FETA**	**FMNKFIYEI**	**0.01% (SB)**
**25**	**Q15007**	**FL2D**	**LQKKYSEEL**	**17%**
26	O60318	GANP	SQNKYIGES	38%
27	Q86SR1	GLT10	HTKKFCFDA	23%
28	Q14789	GOGB1	SLNKYIEEM	1.30%
29	Q9UBN7	HDAC6	HQNKFGEDM	31%
30	P56199	ITA1	STEKFVEEI	13%
31	Q08881	ITK	VAEKYVFDS	41%
32	Q2Q1W2	LIN41	FLNKYGFEG	3.50%
33	P16050	LOX15	VLKKFREEL	11%
34	Q8IWT6	LRC8A	SLKKYSFES	11%
35	O60449	LY75	SLEKYSPDS	27%
36	Q9UPN3	MACF1	LAEKFWYDM	22%
37	Q9UI95	MD2L2	PVEKFVFEI	18%
38	P25103	NK1R	YLKKFIQQV	0.6%(WB)
39	P46459	NSF	ILNKYVGES	14%
40	Q9Y5H3	PCDGA	QLNKYTGEI	5%
41	Q8WUM4	PDC6I	QQKKFGEEI	31%

42	P40855	PEX19	SQEKFFQEL	11%
**43**	**Q9Y2P8**	**RCL1**	**ILNKFIPDI**	**0.8%(WB)**
44	P40937	RFC5	WVEKYRPQT	65%
45	P40937-2	RFC5	MVEKYRPQT	43%
46	Q9UHV5	RPGFL	PTEKFLQEL	36%
47	Q5VT52	RPRD2	SQEKFYPDT	35%
48	Q9H1V8	S6A17	GTKKFMQEL	21%
49	Q8IVG5	SAM9L	MIKKYFEES	28%
50	Q9NRH2	SNRK	LAKKYFAQI	24%
51	P49590	SYHM	LTEKYGEDS	75%
52	Q8WXH0	SYNE2	IQKKYSQQV	13%
53	P42680	TEC	CQNKYPFQV	3%
54	Q8NHR7	TERB2	AMKKFLGEL	5.50%
55	Q93096	TP4A1	TLNKFIEEL	1% (WB)
56	Q12974	TP4A2	TLNKFTEEL	1.3% (WB)
57	Q8WVT3	TPC12	TAEKYFQDV	27%
58	P0CF51	TRGC1	LLEKFFPDV	1.4% (WB)
59	P03986	TRGC2	LLEKFFPDI	3.50%
60	P53804	TTC3	LLEKFVEEC	9.50%
61	Q8TF42	UBS3B	VLKKFAADF	22%
62	P15374	UCHL3	TLKKFLEES	19%
63	Q8IZQ1	WDFY3	PAKKFVFDF	47%
64	A4UGR9	XIRP2	QQKKYLEQL	23%
65	Q9NTW7-4	ZF64B	HMKKFHGDM	37%
66	O43149	ZZEF1	LIEKYVGQF	35%
67	P02768-3	ALBU	FLKKYLYET	0.6%(WB)
68	O75828	CBR3	LMKKFVEDT	18%
69	P33993	MCM7	KVKKFLQEF	30%
70	Q3KQV9	UAP1L	KMEKFVFDV	0.50%
71	Q16222	UAP1	KMEKFVFDI	1.4%(WB)
72	Q14146	URB2	KIEKFTAQF	25%
73	Q9UN37	VPS4A	KVKKFSEDF	44%
74	O75351	VPS4B	KLKKFTEDF	24%
75	P31327	CPSM	EMKKFLEEA	17%
76	Q8WZ42	TITIN	ASNKFGADI	30%
77	O14818	PSA7	EIEKYVAEI	20%
78	Q8IUG5	MY18B	ELEKYVEEL	11%
79	Q92574	TSC1	EQKKYLEDV	32%
80	Q9UGR2	Z3H7B	ESKKYWQQM	70%
81	Q9BXL5	HEMGN	GSEKYSPET	60%
82	P01768	HV330	GSNKYYADS	80%
83	P54132	BLM	KLEKYGAEV	1.10%
84	P14625	ENPL	KSEKFAFQA	22%
85	O75396	SC22B	LSKKYRQDA	85%
86	Q96MZ0	GD1L1	LSKKYWEDG	80%
87	Q5JR59	MTUS2	MSEKFLQEV	8.50%
88	Q9Y5W8	SNX13	NSEKYLEQC	55%

89	Q5BN46	CI116	NSNKFSQQL	32%
90	Q9Y2F5	ICE1	PSEKFGEDL	60%
91	Q53H47	SETMR	TSEKYAQEI	43%
92	Q8TDW4	ST7L	VSEKFSPET	45%
93	P32298	GRK4	YSEKFSEDA	27%
